# The probabilistic termination tool amber

**DOI:** 10.1007/s10703-023-00424-z

**Published:** 2023-05-10

**Authors:** Marcel Moosbrugger, Ezio Bartocci, Joost-Pieter Katoen, Laura Kovács

**Affiliations:** 1 TU Wien, Vienna, Austria; 2 RWTH Aachen University, Aachen, Germany

**Keywords:** Probabilistic programs, Almost sure termination, Martingales, Asymptotic bounds, Recurrence equations

## Abstract

We describe the Amber tool for proving and refuting the termination of a class of probabilistic while-programs with polynomial arithmetic, in a fully automated manner. Amber combines martingale theory with properties of asymptotic bounding functions and implements relaxed versions of existing probabilistic termination proof rules to prove/disprove (positive) almost sure termination of probabilistic loops. Amber supports programs parametrized by symbolic constants and drawing from common probability distributions. Our experimental comparisons give practical evidence of Amber outperforming existing state-of-the-art tools.

## Introduction

Probabilistic programming obviates the need to manually provide inference methods for different stochastic models and enables rapid prototyping [1, 2]. Automated formal verification of probabilistic programs, however, is still in its infancy. With our current work, we provide a step towards closing this gap when it comes to automating the termination analysis of probabilistic programs, which is an active research topic [3–12]. Probabilistic programs are almost-surely terminating (AST) if they terminate with probability 1 on all inputs. They are positively AST (PAST) if their expected runtime is finite [13].

Addressing the challenge of (P)AST analysis, in this paper we describe Amber, a fully automated software artifact to prove/disprove (P)AST. Amber supports the analysis of a class of polynomial probabilistic programs. Probabilistic programs supported in our programming model consist of single loops whose body is a sequence of random assignments with acyclic variable dependencies. Moreover, Amber’s programming model supports programs parametrized by symbolic constants and drawing from common probability distributions, such as *Uniform* or *Normal* (Sect. 3). To automate termination analysis, Amber automates relaxations of various existing martingale-based proof rules ensuring (non-)(P)AST [14] and combines symbolic computation with asymptotic bounding functions (Sects. 4–5). Amber certifies (non-)(P)AST without relying on user-provided templates/bounds over termination conditions. Our experiments demonstrate Amber outperforming the state-of-the-art in automated termination analysis of probabilistic programs (Sect. 7). Our tool Amber is available at https://github.com/probing-lab/amber.

**Related work.** While probabilistic termination is an actively studied research challenge, tool support for probabilistic termination is limited. We compare Amber with computer-aided verification approaches proving probabilistic termination. The tools MGen [4] and LexRSM [7] use linear programming techniques to certify PAST and AST, respectively. A modular approach verifying AST was recently proposed in [11]. Automated techniques for refuting (P)AST were proposed in [8] and techniques for synthesizing polynomial ranking supermartingales using semi-definite programming in [6]. The work [12] introduced a sound and relatively complete algorithm to prove lower bounds on termination probabilities. However, the works of [6, 8, 11, 12] lack full tool support. The recent tools Absynth [15], KoAT2 [16] and ecoimp [17] can establish upper bounds on expected costs, therefore also on expected runtimes, and thus certify PAST. While powerful on respective AST/PAST domains, we note that none of the aforementioned tools support both proving and disproving AST or PAST. Our tool Amber is the first to prove and/or disprove (P)AST in a unifying manner. Our recent work [18] introduced relaxations of existing proof rules for probabilistic (non-)termination together with automation techniques based on *asymptotic bounding functions*. We utilize these proof rule relaxations in Amber and extend the technique of asymptotic bounds to programs drawing from various probability distributions and including symbolic constants.

**Contributions.** This paper describes the tool Amber, a fully automatic open-source software artifact for certifying probabilistic (non-)termination.

- We provide techniques to extend the method of asymptotic bounds for probabilistic termination to support symbolic constants and drawing from common probability distributions which can be continuous, discrete, finitely- or infinitely supported (Sects. 3 and 5).
- We describe the various components and give an overview of the implementation principles of Amber (Sect. 6).
- We extensively compare Amber to related tools and report on our experimental findings (Sect. 7).
- We provide a benchmark suite of 50 probabilistic programs as a publicly available repository of probabilistic program examples (Sect. 7).

**Extensions to **[19]. This paper is an extended version of the Amber tool demonstration paper [19]. Extending [19], we provide the theoretical prerequisites in Sect. 2. Sections 4–5 complement [19] with new material introducing the supported termination proof rules and illustrating Amber’s algorithmic approach towards termination analysis. Moreover, Sect. 5 describes extensions of the asymptotic bound algorithm [18] to programs drawing from common probability distributions and containing symbolic constants. Section 6 goes beyond the details of [19] in describing the different components of Amber and their interplay.

## Preliminaries

By $\mathbb {N}$, $\mathbb {Q}$ and $\mathbb {R}$ we denote the set of natural, rational, and real numbers, respectively. We write $\overline{\mathbb {Q}}$, the real algebraic closure of $\mathbb {Q}$, to denote the field of real algebraic numbers. We write $\overline{\mathbb {Q}}[x_1,\ldots ,x_k]$ for the polynomial ring of all polynomials $P(x_1, \ldots , x_k)$ in *k* variables $x_1,\ldots ,x_k$ with coefficients in $\overline{\mathbb {Q}}$ (with $k\in \mathbb {N}$ and $k\ne 0$). We assume the reader to be familiar with Markov chains and probability theory in general. For more details we refer to [20, 21].

### C-finite recurrences

We recall some relevant notions and results from algebraic recurrences. For more details we refer to [22, 23]. A sequence in $\overline{\mathbb {Q}}$ is a function $f :\mathbb {N}\rightarrow \overline{\mathbb {Q}}$. A recurrence of order *r* for a sequence is an equation $f(i{+}r) = \mathcal {R}(f({i{+}r{-}1}), \dots , f({i{+}1}), f(i), i)$, for some function $\mathcal {R} :\mathbb {R}^{r+1} \rightarrow \mathbb {R}$. A special class of recurrences relevant to our work are *linear recurrences with constant coefficients*, or *C-finite recurrences* in short. A C-finite recurrence for a sequence *f*(*i*) is an equation of the form

1 $$\begin{aligned} f(i{+}r) = a_{r-1} {\cdot } f(i{+}r{-}1) + a_{r-2} {\cdot } f(i{+}r{-}2) + \dots + a_{0} {\cdot } f(i) \end{aligned}$$

where $a_0,\dots ,a_{r-1} \in \overline{\mathbb {Q}}$ are constants and $a_0 \ne 0$. A sequence satisfying a C-finite recurrence (1) is a *C-finite sequence* and is uniquely determined by its initial values $f(0), \dots , f(r{-}1) \in \overline{\mathbb {Q}}$. The terms of a C-finite sequence can be written in closed-form as exponential polynomials (i.e. as a linear combination of exponential sequences and polynomials), depending only on *i* and the initial values of the sequence. That is, if *f*(*i*) is C-finite, then $f(i) = \sum _{n=0}^k P_n(i){\cdot }\lambda _n^i$ where all $P_n(i) \in \overline{\mathbb {Q}}[i]$ and all $\lambda _n \in \overline{\mathbb {Q}}$; we refer to $\sum _{n=0}^k P_n(i) \lambda _n^i$ as an exponential polynomial. Moreover, every polynomial exponential over $i \in \mathbb {N}$ is the solution of some C-finite recurrence. Importantly, closed-forms of C-finite sequences always exist and are computable [23].

Special recurrences relevant for the internals of Amber are inhomogeneous linear recurrences with exponential polynomials as inhomogeneous parts:

2 $$\begin{aligned} f(i{+}1) = a{\cdot }f(i) + \sum _{n=0}^k P_n(i) {\cdot } \lambda _n^i, \end{aligned}$$

where $a \in \overline{\mathbb {Q}}$. Every sequence satisfying a recurrence of form (2) is C-finite, because the inhomogeneous part in (2) is C-finite and all components of systems of C-finite sequences are C-finite. Moreover, if $a \ge 0$ and all $\lambda _n \ge 0$, then the exponential polynomial closed-form for *f*(*i*) only contains positive exponential terms. For such exponential polynomials the limit $l \in \mathbb {R}\cup \{ -\infty , \infty \}$ as $i \rightarrow \infty$ can always be computed [24].

## Amber: Programming model

**Fig. 1:**
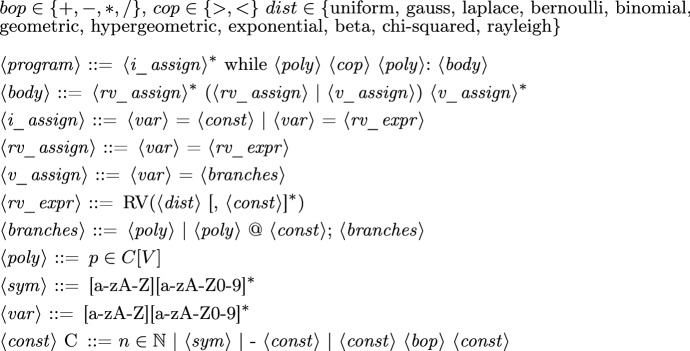
The input syntax of Amber, where *C*[*V*] denotes the set of polynomials in *V* (program variables) with coefficients from *C* (constants); ** is used as the power operator to express polynomials in $\langle poly \rangle$

Amber analyzes the probabilistic termination behavior of a class of probabilistic programs involving polynomial arithmetic and random drawings from common probability distributions, parameterized by symbolic constants. The grammar in Fig. 1 defines the input programs to Amber. Inputs to Amber consist of an initialization part and a while-loop, whose guard is a polynomial inequality over program variables. The initialization part is a sequence of assignments either assigning (symbolic) constants or values drawn from probability distributions. Within the loop body, program variables are updated with either (i) a value drawn from a distribution or (ii) one of multiple polynomials over program variables with some probability. Additional to the structure imposed by the grammar in Fig. 1, input programs are required to satisfy the following *structural constraint: Each variable updated in the loop body only depends linearly and non-negatively on itself and in a polynomial way on variables preceding it in the loop body.* On a high level, this structural constraint is what enables the use of algebraic recurrence relations in probabilistic termination analysis. More concretely, the restriction to linear self-dependencies is necessary to ensure that the resulting recurrence relations (cf. Sect. 5) are C-finite and guaranteed to have computable closed-forms. Even seemingly simple first-order *quadratic* recurrences are problematic: the recurrence $f(n{+}1) = r \cdot f(n)^2 - r \cdot f(n)$ does not have known analytical closed-form solutions for most values of $r \in \mathbb {R}$ [25]. Furthermore, coefficients in linear self-dependencies are required to be non-negative to prevent oscillating dynamics. For instance, the sequence defined by the recurrence $f(n{+}1) = {-}1 \cdot f(n)$ oscillates between 1 and ${-}1$ for $f(0)=1$. Amber computes asymptotic bounds for monomials in program variables using recurrences. A central requirement of the termination analysis technique implemented in Amber (cf. Sect. 5) is that the asymptotic bounds are eventually monotone and non-negative or non-positive. Restricting coefficients in linear self-dependencies to be non-negative ensures this necessary property. Moreover, the algorithm computing asymptotic bounds for a program variable *x* first recursively computes the asymptotic bounds for all (monomials in) program variables on which *x* depends. Hence, to ensure termination, the dependencies among variables must be acyclic. This is guaranteed by restricting variable dependencies to preceding variables.

**Fig. 2:**
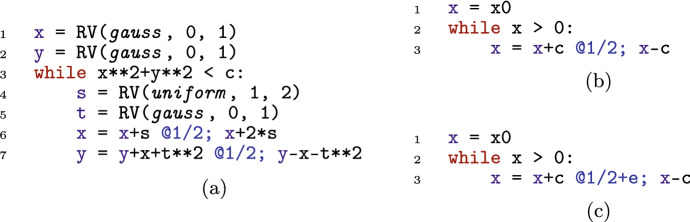
Examples of programs supported by Amber, with symbolic constants $c, x0, e \in \mathbb {R}^+$; program 2a is PAST; program 2b is AST but not PAST; program 2c is not AST

Despite the syntactical restrictions, most existing benchmarks on automated probabilistic termination analysis [18] and dynamic Bayesian networks [26] can be encoded in our programming language. Figure 2 shows three example input programs to Amber. For each of these examples, Amber automatically infers the respective termination behavior, by relying on its workflow described in Sect. 6. Our programming model extends *Prob-solvable loops* [27] with polynomial inequalities as loop guards. For a loop with loop guard $\mathcal {G}$ of the form $P > Q$ we write *G* for the expression $P{-}Q$. In the sequel, we refer to programs of our programming model simply by *loops* or *programs*.

## Proof rules for probabilistic termination

We now describe the theoretical foundations of existing proof rules for establishing probabilistic (non-)termination, which are used and further refined in Amber (Sect. 5).

**Loop space.** Operationally, every program loop represents a Markov chain (MC) with state space $\mathbb {R}^m$ if the loop has *m* program variables. This MC in turn induces a canonical probability space. In this way, every loop $\mathcal {L}$ is associated with a (filtered) probability space $(\Omega ^\mathcal {L}, \Sigma ^\mathcal {L}, (Run_i^\mathcal {L}), \mathbb {P}^\mathcal {L})$. We omit the superscripts if $\mathcal {L}$ is clear from context. The sample space $\Omega$ is the set of all infinite program runs. More precisely, if $\mathcal {L}$ has *m* program variables, then $\Omega = (\mathbb {R}^m)^\omega$. $\Sigma$ is the $\sigma$-algebra constructed from all finite program run prefixes. The purpose of the loop filtration $(Run_i)$ is to capture the information gain as the loop is executed. Every $\sigma$-algebra $Run_i$ of the filtration is constructed from all finite program run prefixes of length $i{+}1$. In this way, $Run_i$ allows measuring events concerned with the first *i* loop iterations. Finally, $\mathbb {P}$ is the probability measure defined according to the intended semantics of the program statements. For a formal definition of $\mathbb {P}$ and more details regarding the semantics of probabilistic loops we refer to [18]. For an expression *E* over the program variables, $E_i$ denotes the random variable mapping a program run to the value of *E* after the *i*-th iteration. With the loop space at hand, the notions of AST and PAST (originally considered in [28]) can be defined in terms of a random variable capturing the termination time.

### Definition 1

The *termination time* of a loop $\mathcal {L}$ with guard $\mathcal {G}$ is the random variable $T^{\lnot \mathcal {G}}$:

$$\begin{aligned} T^{\lnot \mathcal {G}}: \Omega \rightarrow \mathbb {N}\cup \{ \infty \} \quad \text{ with } \quad T^{\lnot \mathcal {G}}(\vartheta ):= \inf \{ i \in \mathbb {N}\mid \vartheta _i \vDash \lnot \mathcal {G}\} \end{aligned}$$

$\mathcal {L}$ is said to be *almost-surely terminating (AST)* if $\mathbb {P}(T^{\lnot \mathcal {G}} < \infty ) = 1$ and *positively almost-surely terminating (PAST)* if $\mathbb {E}(T^{\lnot \mathcal {G}}) < \infty$.

### Termination proof rules

Despite the fact that the problems of AST and PAST are undecidable in general [29], several proof rules—sufficient conditions—have been developed to certify PAST, AST and their negations. On a high level, many proof rules require a witness in the form of an arithmetic expression over program variables that satisfies some conditions based on martingale theory. Amber utilizes three martingale-based proof rules from the literature, one for PAST [4, 5], one for AST [9] and one rule able to certify non-AST and non-PAST [8]. In [18], the authors relaxed these three proof rules such that their conditions only need to hold eventually rather than always. A property *P*(*i*) holds eventually, if *P*(*i*) is true for all $i \ge i_0$ for some $i_0 \in \mathbb {N}$. These relaxations enable using *asymptotic reasoning* when automating the respective proof rules. Amber implements the relaxed versions of these proof rules by choosing the loop guard expression *G* (defined as $P{-}Q$ for loop guard $\mathcal {G} = P > Q$ for polynomials *P* and *Q*) as the potential witness and checking the proof rule conditions using asymptotic bounds (cf. Sect. 5). To certify PAST, Amber uses the *Ranking SM-Rule*.

#### Theorem 1

[Ranking SM-Rule [4, 5, 18]] Let $\mathcal {L}$ be a probabilistic loop with guard $\mathcal {G}$. Assume the following condition holds eventually:

$$\begin{aligned} \mathbb {E}(G_{i+1} - G_i \mid Run_i) \le - \epsilon \text {, for some } \epsilon > 0 \end{aligned}$$

Then, $\mathcal {L}$ is PAST. In this case, *G* is called a *ranking supermartingale*.

Probabilistic programs with an infinite expected runtime can still terminate with probability one. The symmetric one-dimensional random walk (Fig. 2b) is a well-known example that is AST but not PAST. For such programs, the *SM-Rule* provides a solution to certify AST.

#### Theorem 2

[SM-Rule [9, 18]] Let $\mathcal {L}$ be a probabilistic loop with guard $\mathcal {G}$, $d > 0$ and $p \in (0,1]$. Assume the following conditions hold eventually:

1. $$\mathbb {E}(G_{i+1} - G_i \mid Run_i) \le 0$$
2. $$\mathbb {P}(G_{i + 1} - G_i \le -d \mid Run_i) \ge p$$

Then, $\mathcal {L}$ is AST. If *G* satisfies condition 1, it is called a *supermartingale*.

For non-terminating programs, the *Repulsing SM-Rule* can certify their divergence. It is capable of certifying non-AST as well as non-PAST.

#### Theorem 3

[Repulsing SM-Rule [8, 18]] Let $\mathcal {L}$ be a probabilistic loop with guard $\mathcal {G}$. Assume $\forall i: \mathbb {P}(\mathcal {G}_i) > 0$ and that the following conditions hold eventually:

1. $$\mathbb {E}(G_i - G_{i+1} \mid Run_i) \le - \epsilon $$, for some $$\epsilon > 0$$
2. $$|G_i - G_{i+1} |< c$$, for some $$c > 0$$.

Then, $\mathcal {L}$ is *not* AST. If all conditions are true with the domain of $\epsilon$ in condition 1 relaxed to include 0 (i.e. $\epsilon \ge 0$), then $\mathcal {L}$ is *not* PAST.

The *Ranking SM-Rule* as well as the *SM-Rule* require *G*, and the *Repulsing SM-Rule* $-G$, to be a supermartingale. An expression *E* cannot be a supermartingale if $\mathbb {E}(E_{i+1}{-}E_i) > 0$ [18]. The tool Mora [27, 30] can compute an exponential polynomial closed-form of $\mathbb {E}(E_{i+1}{-}E_i)$ for Amber’s input programs. In Amber, we utilize the functionality of Mora to compute a closed-form of $\mathbb {E}(G_{i+1}{-} G_i)$. Amber uses this closed-form in trying to rule-out the applicability of some of the proof rules.

## Effective termination analysis through asymptotic bounds

The conditions in the proof rules from Sect. 4.1 contain three *types of inequalities*:

- **Type 1:** Inequalities over conditional expected values, as $\mathbb {E}(G_{i+1}{-}G_i \mid Run_i) \le - \epsilon$ ($\le 0$) in the *Ranking SM-Rule* (*SM-Rule*) for proving PAST (AST).
- **Type 2:** Inequalities over conditional probabilities, as $\mathbb {P}(G_{i + 1}{-}G_i \le -d \mid Run_i) \ge p$ in the *SM-Rule* for establishing AST.
- **Type 3:** Inequalities over absolute values, as $|G_i{-}G_{i+1} |< c$ in the *Repulsing SM-Rule* for disproving AST.

In the sequel we detail how these three type of inequalities are handled in Amber for proving/disproving (P)AST.

**Type 1.** For Amber’s programming model, the expression $\mathbb {E}(G_{i+1}{-}G_i \mid Run_i)$ is a polynomial in the program variables. For the program in Fig. 2a we have $G = c {-} x^2 {-} y^2$. The expression $\mathbb {E}(G_{i+1}{-}G_i \mid Run_i) = \mathbb {E}(G_{i+1} \mid Run_i){-}G_i$ can be computed by starting with *G*, substituting left-hand sides of assignments by right-hand sides in a bottom-up fashion, averaging over probabilistic statements and finally subtracting *G*. For Fig. 2a, this leads to the polynomial $\mathbb {E}(G_{i+1}{-}G_i \mid Run_i) = {-}x_i^2 {-} 11x_i {-} \nicefrac {115}{6}$. Thus, the expected change of the loop guard from an arbitrary iteration *i* to iteration $i{+}1$ is ${-}x_i^2 {-} 11x_i {-} \nicefrac {115}{6}$, where $x_i$ is the value of program variable *x* after iteration *i*. For an input program and a polynomial *poly*, $\mathbb {E}(poly_{i+1} \mid Run_i)$ itself is always a polynomial. That is because all expressions in probabilistic branching statements are polynomials, all branching probabilities are constants and all distributions input programs can draw from have constant parameters and thus also constant moments. Crucially, all inequalities in the termination proof rules only need to hold eventually. Therefore, knowing the asymptotic behavior of the polynomial $\mathbb {E}(G_{i+1}{-}G_i \mid Run_i)$ can be helpful in answering the respective inequalities: for instance, an asymptotic upper bound to $\mathbb {E}(G_{i+1}{-} G_i \mid Run_i)$ that tends to a negative number witnesses that eventually $\mathbb {E}(G_{i+1}{-}G_i \mid Run_i) \le - \epsilon$ for some $\epsilon > 0$.

**Type 2.** After fixing the values drawn from distributions in the loop body at iteration *i*, every expression, and in particular *G*, can only progress to finitely many expressions in iteration $i{+}1$. We refer to these possible follow-up expressions, as *branches*. For the program in Fig. 2b, the expression *G* ($=x$) is either $x{+}c$ or $x{-}c$ after one iteration. If for at least one of these branches *B* of *G*, we have that eventually $B_i{-}G_i \le -d$ for some $d > 0$ for any choice of values drawn from distributions, then it holds that $\mathbb {P}(G_{i + 1}{-}G_i \le -d \mid Run_i) \ge p$ for some $p > 0$. This holds, due to the fact that all probabilities in probabilistic branching statements are constant and non-zero. Similar to the inequalities of type 1, asymptotic bounds provide a method to answer inequalities of type 2: if for some branch *B* of *G* and any choice of values drawn from probability distributions, the polynomial $B_i {-}G_i$ obeys an asymptotic upper bound tending to a negative number, then it holds that eventually $\mathbb {P}(G_{i + 1}{-}G_i \le -d \mid Run_i) \ge p$ for some $d > 0$ and $p \in (0,1]$.

**Type 3.** In contrast to the inequalities of type 2 that have to hold with at least some non-zero probability, inequalities of type 3 have to hold almost-surely, that means with probability one. Nevertheless, type 3 inequalities can be approached similarly as type 2 inequalities: if for *every* (in contrast to *some* as for type 2 inequalities) branch *B* of *G* and any choice of values drawn from probability distributions, eventually $|G_i {-} B_i |< c$ for some $c > 0$, then eventually and almost-surely $|G_{i} {-} G_{i+1} |< c$. In contrast to type 1 and 2 inequalities, tackling type 3 inequalities with asymptotic bounds requires one extra step. Due to the presence of the absolute value function, asymptotic upper bounds for the polynomials $G_i {-} B_i$ do not suffice. Additional to upper bounds, asymptotic lower bounds are needed. Given an asymptotic upper bound *u*(*i*) and an asymptotic lower bound *l*(*i*) for the polynomial $G_i {-} B_i$, $\max (-l(i), u(i))$ is an asymptotic upper bound for $|G_i {-} B_i |$.

### Computing asymptotic bounds

We argued that all main conditions of the termination proof rules from Sect. 4.1 reduce to the task of finding asymptotic lower- and upper bounds for polynomials in program variables. For this purpose, Amber utilizes a recently introduced *bound algorithm* [18]. The algorithm builds on the notion of dominant functions.

#### Definition 2

Let *f* and *g* be two functions from $\mathbb {N}$ to $\mathbb {R}$. We say *f* *dominates* *g* if eventually $c {\cdot } f(i) \ge g(i)$ for some $c \in \mathbb {R}^+$. Let *F* be a finite set of functions from $\mathbb {N}$ to $\mathbb {R}$. A function $f \in F$ is *most dominant* with respect to *F*, if *f* dominates all functions in *F*. Similarly, *f* is *least dominant* with respect to *F*, if every $g \in F$ dominates *f*.

The bound algorithm described in this section produces bounds in the form of exponential polynomials with positive exponential terms as mentioned in Sect. 2. For every such function *f*, we can always construct a monotonic and non-positive or non-negative function *g* with the same asymptotic behavior, meaning that *g* dominates *f* and *f* dominates *g*. The function *g* can be established by simplifying *f* to its fastest increasing or decreasing term, its *leading term*. For instance, if $f(i) = i2^i - 2^i - i^2$, then $g(i) = i2^i$ is monotonic, non-negative, and has the same asymptotic behavior as *f*. In the remainder, we assume that every asymptotic lower- and upper bound is simplified to its leading term. Moreover, for two exponential polynomials with positive exponential terms, we can always decide which dominates the other by comparing their leading terms. We illustrate the algorithm for computing asymptotic bounds in the following example. For the algorithm’s pseudo-code and further details, we refer to [18].

#### Example 1

Consider the following program:
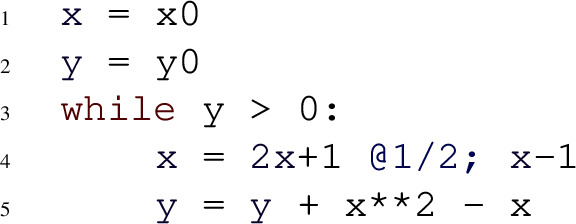


Assume, we want to compute an asymptotic lower bound and asymptotic upper bound for the program variable *y*. This means that we are trying to find functions *l*(*i*) and *u*(*i*) such that eventually and almost-surely $c_1 {\cdot } l(i) \le y_i \le c_2 {\cdot } u(i)$ for some positive constants $c_1$ and $c_2$. For every iteration *i*, $y_{i+1}$ is either equal to $y_i {+} 4x_i^2 {+} 2x_i$ or equal to $y_i {+} x_i^2 {-} 3x_i {+} 2$, both with probability $\nicefrac {1}{2}$. These polynomials are the branches of *y*. The algorithm in [18] first recursively computes asymptotic lower- and upper bounds for the monomials *x* and $x^2$ in order to construct bounds for *y*.

**Asymptotic bounds for **${\varvec{x}}:$ The branches of *x* are $2x_i{+}1$ and $x_i{-}1$ with inhomogeneous parts 1 and $-1$ respectively. The bound algorithm first computes bounds for the inhomogeneous parts. Because the inhomogeneous parts are both constants, both their lower- and upper bounds are just given by the inhomogeneous parts themselves. This is the base case of the algorithm. The base case will always be reached, because of the constraint of Amber’s programming model that the dependencies among program variables in the loop body are acyclic (cf. Sect. 3). The *recurrence coefficients* of *x* are 2 and 1 respectively. These are the constant coefficients of $x_i$ in the branches of *x*. The results of [18] establish that an upper bound for *x* is given by the solution of one of the following four recurrence relations:

3 $$\begin{aligned} f(i+1) = a {\cdot } f(i) + 1, \text { for } f(0) \in \{d, -d\}, a \in \{2, 1\} \end{aligned}$$

The recurrences (3) are inhomogeneous first-order recurrences. Their recurrence coefficients are given by the minimum and maximum recurrence coefficients of *x*. The inhomogeneous term in (3) is the most dominant upper bound of the inhomogeneous parts of *x*. The initial values of the recurrences (3) are *d* or $-d$ for a positive symbolic constant *d*. With a simple static analysis, Amber establishes that the program variable *x* can become positive as well as negative. Because *x* can be positive, *d* is among the initial values, and because *x* can be negative $-d$ is also required as an initial value. The solutions (closed-forms) to the four recurrences of *f*(*i*) are respectively given by

- $$(d{+}1)2^i{-}1$$;
- $$(1{-}d)2^i{-}1$$;
- $$i{+}d$$;
- $$i{-}d$$.

According to [18], one of these four solutions is an upper bound to *x*. The closed form $(d{+}1)2^i{-}1$ of *f*(*i*) dominates all other solutions. As we are only interested in asymptotic bounds modulo a constant factor, an asymptotic upper bound for *x* is therefore given by the *leading term* of $(d{+}1)2^i{-}1$; that is, *x* is asymptotically upper bounded by $2^i$.

An asymptotic lower bound for *x* is computed analogously as the *least* dominant solution to one of the recurrences

4 $$\begin{aligned} f(i+1) = a {\cdot } f(i) - 1, \text { for } f(0) \in \{d, -d\}, a \in \{2, 1\}. \end{aligned}$$

In contrast to the upper bound computation, the inhomogeneous term in (4) is given by the *least dominant lower bound* of the inhomogeneous parts of *x*, i.e. ${-}1$. The leading term in the least dominant solution of the recurrences (4) is $-2^i$ and provides an *asymptotic lower bound* for *x*. Consequently, we established that eventually and almost surely

$$\begin{aligned} c_1 {\cdot } (-2^i) \le x_i \le c_2 {\cdot } 2^i \text { for some } c_1, c_2 \in \mathbb {R}^+. \end{aligned}$$

An absolute bounding function of *x* is an asymptotic bound for $|x_i |$ and is given by the most dominant function of $u(i)=2^i$ and $-l(i) = -(-2^i)$. Note, that all recurrences in (3)–(4) are first-order inhomogeneous linear recurrences with non-negative coefficients and exponential polynomials as inhomogeneous parts such that all exponential terms are positive. As argued in Sect. 2, recurrences of this type can always be solved automatically and lead to solutions for which their limits can be computed.

**Asymptotic bounds for **${\varvec{x}^2}$: The branches of the monomial $x^2$ are $4x^2 {+} 4x {+} 1$ and $x^2 {-} 2x {+} 1$. Therefore the recurrence coefficients are given by 4 and 1. The inhomogeneous parts are $4x{+}1$ and ${-}2x{+}1$. Utilizing the already computed bounds for *x*, we get that $4x{+}1$ as well as $-2x+1$ are asymptotically upper bounded by $2^i$ and lower bounded by $-2^i$. Hence, the most dominant upper bound of the inhomogeneous parts is $2^i$ and the least dominant lower bound is $-2^i$. Following the bound algorithm of [18], we get that an asymptotic upper bound is given by the most dominant solution of the following recurrences:

$$\begin{aligned} f(i+1) = a {\cdot } f(i) + 2^i, \text { for } f(0) \in \{d\}, a \in \{4, 1\} \end{aligned}$$

Computing the solutions of these recurrences and taking the leading term of the most dominant solution leads to the asymptotic upper bound $4^i$ for $x^2$. Note that the possible initial values are restricted to the positive constant *d*, as $x^2$ can never be negative. An asymptotic lower of $-2^i$ can be computed analogously. However, due to the non-negativity of $x^2$, the constant 0 is a tighter lower bound which is taken into account by the bound algorithm.

**Asymptotic bounds for **${\varvec{y}}$: Finally, we can compute asymptotic bounds for *y* using the bounds for *x* and $x^2$. The branches of *y* are $y {+} 4x^2 {+} 2x$ and $y {+} x^2 {-} 3x {+} 2$ with inhomogeneous parts $4x^2 {+} 2x$ and $x^2 {-} 3x {+} 2$, respectively. Moreover, *y* has a single recurrence coefficient of 1. An asymptotic upper bound for the inhomogeneous part $x^2 {-} 3x {+} 2$ can be established by substituting the bounds for the individual monomials. For $x^2$ we substitute its upper bound and for *x* its lower bound, due to the negative coefficient of *x* in the respective branch. For $x^2 {-} 3x {+} 2$ this leads to an asymptotic upper bound of $4^i$ and an asymptotic lower bound of $-2^i$. Likewise, for the inhomogeneous part $4x^2 {+} 2x$, we get an asymptotic upper bound of $4^i$ and an asymptotic lower bound of $-2^i$. Therefore, the most dominant upper bound of the inhomogeneous parts is $4^i$, and the least dominant lower bound is $-2^i$. Similar to the bounds computations for *x* and $x^2$, an asymptotic upper bound is given by the most dominant solution of

$$\begin{aligned} f(i+1) = a {\cdot } f(i) + 4^i, \text { for } f(0) \in \{d, -d\}, a \in \{1\}. \end{aligned}$$

The leading term of the most dominant solution is $4^i$ and represents an asymptotic upper bound for *y*. An asymptotic lower bound for *y* of $-2^i$ can be computed analogously.

The bound algorithm introduced in [18] only supports programs of Amber’s programming model, where every assignment in the loop body is a probabilistic branching statement over polynomials. In the remainder of this section, we describe how the techniques of [18] can be extended to support symbolic constants and drawing from common probability distributions with constant parameters.

### Supporting symbolic constants

A symbolic constant represents an arbitrary number from an infinite set of real numbers. For example, the program in Fig. 2b encodes a symmetric one-dimensional random walk with symbolic step size *c*. For our purposes, defining a symbolic constant *c* to semantically represent *any* arbitrary real number $c \in \mathbb {R}$ is problematic, as illustrated in the following example.

#### Example 2

Consider the following program with symbolic constant *c*:
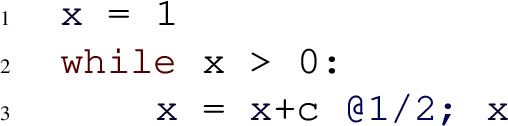


Following the bound algorithm of [18] for *x* would result in the lower bound of *x* being the least dominant of $c {\cdot } i$ and 1. Now, if *c* semantically represents an *arbitrary* real number, we cannot conclusively decide whether $c \cdot i$ or 1 is more dominant: if $c > 0$, then $c{\cdot }i$ dominates 1 and if $c \le 0$, then 1 dominates $c{\cdot }i$.

To remedy the problem illustrated in the previous example, Amber adopts the semantic that symbolic constants represent an arbitrary *positive* real number. Negative constants can be modeled with the explicit use of “−”. Still, the bound algorithm is incomplete for input programs with positive symbolic constants. A counter-example can be constructed from Example 2 by replacing *c* with $c{-}d$ where both *c* and *d* are symbolic constants. Now, the lower bound for the variable *x* is the least dominant of $(c{-}d){\cdot }i$ and 1 which cannot be answered without a case distinction involving the symbolic constants *c* and *d*. Nevertheless, experiments show that adopting the semantic of positive symbolic constants is useful and provides a solution to many challenging benchmarks (cf. Sect. 7).

### Supporting common probability distributions

Amber supports programs drawing from various common probability distributions with constant parameters (cf. Fig. 1). The first key property of every supported distribution $\mathcal {D}$ is that $\mathbb {E}(\mathcal {D}^p)$ *exists and is computable for every *$p \in \mathbb {N}$. This ensures that for any polynomial *poly* in program variables, $\mathbb {E}(poly_{i+1} \mid Run_i)$ remains a polynomial.

The second key property is that $\mathcal {D}$’*s* *support is an interval*. More precisely, if $\mathcal {D}$ is continuous, then $supp (\mathcal {D}) = (a,b)$ (or [*a*, *b*]) for $a,b \in \mathbb {R}\cup \{-\infty , \infty \}$ and if $\mathcal {D}$ is discrete, then $supp (\mathcal {D}) = \{a, a{+}1, \dots , b{-}1, b\}$ for $a,b \in \mathbb {R}\cup \{-\infty , \infty \}$. Because the support of $\mathcal {D}$ is an interval, tight bounds for the support of $\mathcal {D}^p$ for $p \in \mathbb {N}$ can be computed using interval arithmetic.

Amber extends the main bound algorithm to support programs drawing from such distributions. Let *x* be a program variable drawing from a probability distribution, *M* a monomial of program variables not containing *x*, and $p \in \mathbb {N}$. Then Amber computes the asymptotic bounds for the monomial $x^p {\cdot } M$ in the following way: First, an upper bound *u*(*i*) and lower bound *l*(*i*) for *M* are computed recursively. Second, the boundaries *a* and *b* (with $a \le b$) of the support of $x^p$ are computed using interval arithmetic. Finally, an upper bound (lower bound) of $x^p \cdot M$ is given by the most dominant function (least dominant function) of $a {\cdot } u(i)$, $b {\cdot } u(i)$, $a {\cdot } l(i)$ and $b {\cdot } l(i)$. Due to Amber supporting unbounded distributions, *a*, *b*, *l*(*i*) and *u*(*i*) can be $\pm \infty$. The handle calculations involving infinities, we use the usual arithmetic rules for $\pm \infty$: $x {+} \infty {=} \infty$; $x {-} \infty {=} {-}\infty$; if $x > 0$ then $x {\cdot } \infty {=} \infty$; if $x < 0$ then $x {\cdot } \infty {=} {-} \infty$. Note that, because the asymptotic bounds *b*(*i*) are always monotonic and non-positive or non-negative, Amber can always decide whether $\infty \cdot b(i)$ is $\infty$ or $-\infty$ (if *b*(*i*) itself is not $\pm \infty$ or 0). In case of indeterminate forms ($\infty {-} \infty$ and $0 {\cdot } \infty$), Amber aborts the bound computation and resorts to the loosest possible bounds of $-\infty$ and $\infty$.

#### Example 3

Let *x* be a program variable drawing from a continuous uniform distribution between $$-1$$ and 2 and *M* a monomial of program variables not containing *x*. Assume *M* obeys an asymptotic lower bound $$l(i) = i$$ and an asymptotic upper bound $$u(i) = i^2$$. Asymptotic bounds for $$x^3 {\cdot } M$$ are computed as follows. We have $$ supp (x^3) = (-1,8)$$. Let $$F = \{-1 {\cdot } i^2, 8 {\cdot } i^2, -1 {\cdot } i, 8 {\cdot } i\}$$. The most dominant function in *F* is $$8 {\cdot } i^2$$ and because positive constant factors of asymptotic bounds can be absorbed, $$i^2$$ is an asymptotic upper bound for $$x^3 {\cdot } M$$.

Although Amber requires the parameters of distributions to be constant, some state-dependent parameters can be modeled through distribution transformations. For instance, $\text {Normal}(poly, c)$ is equivalent to $poly + \text {Normal}(0,c)$. Likewise, $\text {Uniform}(poly_1, poly_2)$ is equivalent to $poly_1{+}(poly_2{-}poly_1) {\cdot } \text {Uniform}(0,1)$ for the continuous uniform distribution. Similar transformations exist for other distributions.

With the generalized bound algorithm, Amber can compute asymptotic upper- and lower bounds for polynomials of program variables, even if the programs draw from probability distributions. However, this generalization alone is not sufficient, in particular for the *SM-Rule*.

#### Example 4

The following program models a symmetric 1-dimensional random walk:
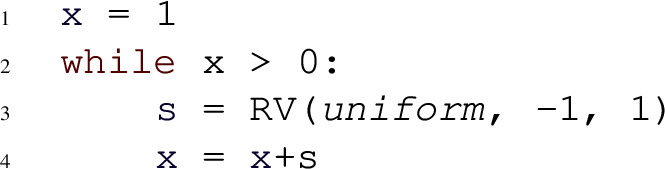


The program can be proven to be AST using the *SM-Rule*. We have $G = x$. Amber computes $\mathbb {E}(x_{i+1} {-} x_i \mid Run_i) = 0$ and hence establishes condition 1 of the *SM-Rule*. However, condition 2 poses a problem. Amber extracts the only branch of *x*, that is $x{+}s$, and computes the asymptotic bounds for $x{+}s - x = s$, resulting in the lower bound $-1$ and the upper bound 1. Because the upper bound is always positive, without further information Amber cannot conclude that *x* decreases by some constant with constant probability. For this example, the problem can be mitigated by constructing an equivalent program in which the variable *s* is split into three different parts:
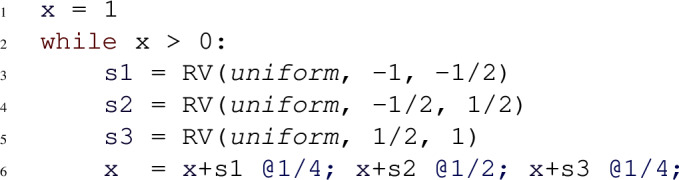


Now, for the branch $x{+}s1$, Amber established the bounds for $x{+}s1 - x = s1$ to be $-1$ and $\nicefrac {-1}{2}$. As the asymptotic upper bound is negative, Amber concludes that eventually *x* decreases by at least some constant with at least some constant probability. Thus, condition 2 of the *SM-Rule* is verified and Amber certifies the program to be AST.

In Example 4, the program variable *s* is drawn from a uniform distribution whose support contains positive and negative values. Without further information, Amber can only establish that *s* is lower bounded by $-1$ and upper bounded by 1 but is oblivious to the fact that there is a constant probability such that *s* is negative. In Example 4, this fact is made explicit to Amber through constructing an equivalent program by splitting the uniform distribution into three different parts such that two parts are bounded away from 0. In general, this exact approach is not feasible when drawing from more complex distributions. However, note that neither the exact probability of $\nicefrac {1}{4}$ of branch $x{+}s1$, nor the exact distribution of *s*1 are necessary to answer condition 2 of the *SM-Rule*. It suffices that the branch is associated with *some* constant positive probability and that the support of *s*1 is strictly negative and bounded away from 0. In this sense, the only relevant information about the distribution of *s* is its support.

Following this observation, Amber implements an over-approximation when considering the branches of expressions, abstracting from concrete distributions: let *B* be a branch containing a variable *s* drawn from a probability distribution $\mathcal {D}$ with support boundaries $a < 0$ and $b > 0$. With $Tr(\mathcal {D}; \alpha , \beta )$ we denote the *truncated distribution* of $\mathcal {D}$ with lower bound $\alpha$ and upper bound $\beta$. Assume $\mathcal {D}$ is a continuous distribution. For discrete distributions, the following process is analogous. Let $\epsilon > 0$ with $|a |> \epsilon$ and $b > \epsilon$ and define

$$\begin{aligned} p_1:= \int _a^{-\epsilon } d \mathcal {D} p_2:= \int _{-\epsilon }^\epsilon d \mathcal {D} p_3:= \int _\epsilon ^b d \mathcal {D}. \end{aligned}$$

We have $p_1, p_2, p_3 > 0$ and can split $\mathcal {D}$ into three different parts such that one part has strictly negative, one part strictly positive support, and both supports are bounded away from 0. With $C \sim Categorical(3, p_1, p_2, p_3)$ we have

$$\begin{aligned}&s \sim \, [C=1] \cdot Tr(\mathcal {D}, a, -\epsilon ) + \\&\quad [C=2] \cdot Tr(\mathcal {D}, -\epsilon , \epsilon ) + \\&\quad [C=3] \cdot Tr(\mathcal {D}, \epsilon , b). \end{aligned}$$

[*P*] denotes the Iverson bracket which equals 1 if *P* is true and 0 otherwise. Now, the goal of Amber is to split the branch *B* containing *s* into three branches, where *s* is replaced by $s_1 \sim Tr(\mathcal {D}, a, -\epsilon )$, $s_2 \sim Tr(\mathcal {D}, -\epsilon , \epsilon )$ and $s_3 \sim Tr(\mathcal {D}, \epsilon , b)$ respectively. However, the distributions of $s_1$, $s_2$, and $s_3$ are potentially more complex than the original distribution $\mathcal {D}$, and the constants $p_1$, $p_2$, and $p_3$ each require solving an integral. Amber overcomes these issues with over-approximation. As previously argued, the precise values of $p_1$, $p_2$, and $p_3$ are not needed and only required to be positive, which is guaranteed. Moreover, the only relevant information about the distributions of $s_1$, $s_2$, and $s_3$ are their supports. Therefore, Amber over-approximates $Tr(\mathcal {D}, \alpha , \beta )$ by $Symb(\alpha , \beta )$, where $Symb(\alpha , \beta )$ represents any distribution $\mathcal {D}'$ with $supp (\mathcal {D}') = [\alpha , \beta ]$. With $v \sim Symb(\alpha , \beta )$ we denote that $v \sim \mathcal {D}'$ for some $\mathcal {D}' \in Symb(\alpha , \beta )$. Consequently, for condition 2 of the *SM-Rule* and for condition 2 of the *Repulsing SM-Rule*, Amber splits every branch *B* containing a variable *s* drawn from a probability distributions $\mathcal {D}$ with mixed-sign support into three new branches $B[s/s_1]$, $B[s/s_2]$, and $B[s/s_3]$. The substituted variables are such that $s_1 \sim Symb(a, -\epsilon )$, $s_2 \sim Symb(-\epsilon , \epsilon )$, and $s_3 \sim Symb(\epsilon , b)$ where *a* and *b* are the boundaries of the support of $\mathcal {D}$ and $\epsilon$ is a fresh positive symbolic constant. This process is repeated until all such variables *s* have been eliminated and the only distributions with mixed-sign supports left are over-approximations.

#### Example 5

Consider the following program:
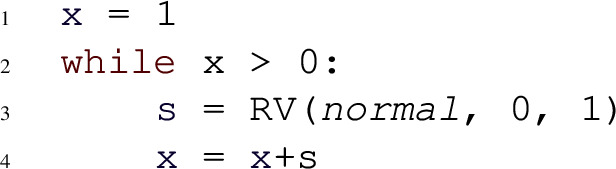


For $G = x$, Amber computes the expression $\mathbb {E}(x_{i+1} {-} x_i \mid Run_i) = 0$. Therefore, condition 1 of the *SM-Rule* is satisfied. Regarding condition 2, Amber starts with the only branch of *x* which is $x{+}s$. The branch $x{+}s$ contains the variable *s* whose distribution has the mixed-sign support $(-\infty , \infty )$. Hence, Amber splits the branch $x{+}s$ into the three branches (1) $x{+}s_1$, (2) $x {+} s_2$, and (3) $x {+} s_3$, where $s_1 \sim Symb(-\infty , -\epsilon )$, $s_2 \sim Symb(-\epsilon , \epsilon )$, $s_3 \sim Symb(\epsilon , \infty )$ and $\epsilon$ is a fresh positive symbolic constant. For the new branch (1) $x {+} s_1$, an upper bound for $x {+} s_1 - x = s_1$ is given by $-\epsilon$. Therefore, *x* decreases by at least $\epsilon$ with some constant positive probability, confirming that also condition 2 of the *SM-Rule* is satisfied. Consequently, Amber certifies AST for this example.

## Amber: Implementation and components

**Fig. 3:**
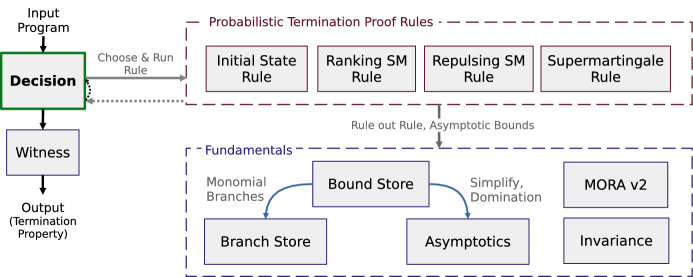
Main components of Amber and interactions between them

**Implementation.** Amber is implemented in python3 and relies on the lark-parser^1^ package to parse its input programs. Further, Amber uses the diofant^2^ package as its computer-algebra system to (i) construct and manipulate mathematical expressions symbolically; (ii) solve algebraic recurrence relations, and (iii) compute function limits. To compute closed-form expressions for statistical moments of monomials over program variables only depending on the loop counter, Amber uses the tool Mora [27]. However, for efficient integration within Amber, we reimplemented and adapted the Mora functionalities exploited by Amber (Mora v2), in particular by deploying dynamic programming to avoid redundant computations. Altogether, Amber consists of $\sim 2000$ lines of code. In what follows we discuss the main components of Amber, as illustrated in Fig. 3.

### Decision in Amber

After parsing the input program, the *decision* module of Amber is executed to initialize and call the probabilistic termination proof rules to be used on the input program. In order to initialize the proof rules, Amber’s *decision* module first constructs three expressions: (1) $\mathbb {E}(G_{i+1} {-} G_i \mid Run_i)$ (martingale expression); (2) ${\mathbb {E}(G_{i} {-} G_{i+1} \mid Run_i)}$ (negated martingale expression); and (3) $\mathbb {E}(G_{i+1} {-} G_i)$ (expected loop guard change). For Fig. 2a with loop guard $x^2 {+} y^2 < c$, we get the following expressions: (1) $\mathbb {E}(G_{i+1} {-} G_i \mid Run_i) = {-}x_i^2 {-} 11x_i {-} \nicefrac {115}{6}$; (2) ${\mathbb {E}(G_{i} {-} G_{i+1} \mid Run_i)} = x_i^2 {+} 11x_i {+} \nicefrac {115}{6}$; and (3) $\mathbb {E}(G_{i+1} {-} G_i) = {-}(\nicefrac {81}{16}) i^2 {-} (\nicefrac {1225}{48}) i {-} \nicefrac {121}{6}$. Amber utilizes the relaxed proof rules from Sect. 4 and automates them using asymptotic bounds (cf. Sect. 5). As such, the *decision* module of Amber initializes relaxed proof rules with the expressions above, applies the respective proof rules to the input program, and reports the analysis result containing potential witnesses for (non-)PAST or (non-)AST.

### Probabilistic termination proof rules in Amber

**Initial state rule.** The *Initial State Rule* checks whether or not the initial state, given by the assignments preceding the loop, already falsifies the loop guard. More precisely, the rule returns a witness for PAST if the initial state falsifies the loop guard with probability one. The rule considers all possible combinations of lower and upper bounds of the initial assignments to the variables given by the support of the respective distributions.

#### Example 6

In Fig. 2a, the symbolic constant *c* in the loop guard represents an arbitrary positive constant. Therefore, for Fig. 2a the probability of the initial state falsifying the loop guard is not 1 and the *Initial State Rule* does not return a witness for PAST.

**Ranking SM-rule.** The *Ranking SM-Rule* checks whether the polynomial *G* is eventually a ranking supermartingale (i.e. $\mathbb {E}(G_{i+1} {-} G_i \mid Run_i) \le -\epsilon$) to conclude the input program to be PAST. If ${\mathbb {E}(G_{i+1} {-} G_i) > 0}$, *G* cannot be a (ranking) supermartingale. The rule determines its own applicability using diofant and the expected loop guard change $\mathbb {E}(G_{i+1} {-} G_i)$ to check ${\mathbb {E}(G_{i+1} {-} G_i) > 0}$. If the rule is applicable, the *Bound Store* module of Amber is called to compute an asymptotic upper bound *u*(*i*) for the martingale expression $\mathbb {E}(G_{i+1} {-} G_i \mid Run_i)$ (see Sect. 6.3). If $\lim _{i \rightarrow \infty } u(i) < 0$, then *G* is eventually a ranking supermartingale and the input program is PAST. The *Ranking SM-Rule* uses diofant to verify $\lim _{i \rightarrow \infty } u(i) < 0$. If the condition holds, the *Ranking SM-Rule* constructs and returns a witness for PAST.

#### Example 7

For Fig. 2a, we have $\mathbb {E}(G_{i+1} {-} G_i) = -(\nicefrac {81}{16}) i^2 - (\nicefrac {1225}{48}) i - \nicefrac {121}{6} \not > 0$. Thus the *Ranking SM-Rule* is applicable. For the martingale expression $\mathbb {E}(G_{i+1} {-} G_i \mid Run_i) = -x_i^2 - 11x_i - \nicefrac {115}{6}$, the *Bound Store* module computes an upper bounding function $u(i) = -i^2$. Because $\lim _{i \rightarrow \infty } u(i) = - \infty < 0$, the *Ranking SM-Rule* returns the martingale expression together with *u*(*i*) as a witness for Fig. 2a being PAST.

**SM-rule.** If the *Ranking SM-Rule* fails, the *SM-Rule* attempts to certify AST. The rule checks whether *G* is eventually a supermartingale (i.e. $\mathbb {E}(G_{i+1} {-} G_i \mid Run_i) \le 0$) and whether *G* eventually decreases at least by some fixed constant with positive probability. The applicability criterion for the proof rule is the same as for the *Ranking SM-Rule* ($\mathbb {E}(G_{i+1} {-} G_i) \not > 0$), implemented in the same way. Moreover, Amber concludes *G* to be a supermartingale similarly to concluding it to be a ranking supermartingale. The only difference is that for the martingale expression’s upper bound *u*(*i*), its limit is allowed to be 0 (instead of negative). Amber automates the decrease condition by looping through all branches of *G*, splitting them as described in Sect. 5.3, and checking whether for one of the resulting branches *B*, the polynomial $B - G$ has an upper bounding function with a negative limit. This entails that eventually *G* decreases in any iteration with positive probability.

#### Example 8

For Fig. 2b, we have $G = x$. The martingale expression is $\mathbb {E}(G_{i+1} {-} G_i \mid Run_i) = 0$, which has limit 0 implying that *G* is a supermartingale. Amber retrieves the two branches of *G*, namely $x {+} c$ and $x {-} c$, where *c* is a positive symbolic constant. For the second branch Amber computes $x {-} c - x = -c$ which it determines to have the negative limit $-c$. Therefore, Amber concludes that *G* (eventually) decreases by (at least) $-c$ with positive probability and returns the martingale expression, the eventually decreasing branch and its asymptotic bound as a witness for AST.

**Repulsing SM-rule.** The *Repulsing SM-Rule* can potentially certify non-AST and non-PAST. It is applied in Amber whenever either the status of AST or PAST of the input program is not yet known after applying the *Ranking SM-Rule* and the *SM-Rule*. Moreover, $\mathbb {E}(G_{i+1} {-} G_i) \not < 0$ has to hold in order for the rule to be applicable because $-G$ needs to be a (ranking) supermartingale to certify non-PAST (non-AST). The applicability criterion as well as checking $-G$ to be a (ranking) supermartingale is realized with the same techniques as for the aforementioned proof rules. Additionally, Amber has to verify two more properties: (i) Eventually $|G_i {-} G_{i+1} |< c$ for some $c \in \mathbb {R}^+$; and (ii) in every iteration, there is a positive probability of *G* not decreasing. The first property (i) is realized with retrieving an absolute bounding function *a*(*i*) from the *Bound Store* module and checking whether *a*(*i*) is dominated by 1. Amber verifies the property (ii) by looping through all branches of *G*, splitting them as described in Sect. 5.3, and checking whether for one of the resulting branches *B*, the expression $G{-}B$ is always non-negative, with a simple static analysis. This entails that there is always a positive probability that *G* does not decrease. Amber returns a witness for non-PAST (non-AST) if all properties are satisfied and $-G$ is a (ranking) supermartingale.

#### Example 9

For Fig. 2c with $-G = -x$ we have the negative martingale expression $\mathbb {E}(G_i {-} G_{i+1} \mid Run_i) = -2c{\cdot }e$, where *c* and *e* are positive symbolic constants. Therefore, $-G$ is a ranking supermartingale. The two branches of $-x$ are (1) $-x {-} c$ and (2) $-x {+} c$. For both branches *B* we have $|B {-} (-x) |= c$ and a corresponding absolute bounding function $a(i) = c$. Hence, property (i) is satisfied. Property (ii) holds, because there is always a possibility of *G* not decreasing through branch (2). Thus, for Fig. 2c as input, the *Repulsing SM-Rule* returns a witness for non-AST.

### Fundamentals in Amber

**Bound store.** Amber’s *Bound Store* component derives lower, upper and absolute bounds for polynomials over program variables. These bounds are used by Amber’s termination proof rules (cf. Sect. 6.2). Asymptotic bounding functions only depend on the loop counter *i* and asymptotically bound the value of a program variable polynomials (modulo a positive constant factor). Asymptotic bounding functions for polynomials arise from combining bounding functions of its monomials. For monomials, asymptotic bounding functions are computed using the bound algorithm introduced in Sect. 5.

**Other fundamentals.** The *Branch Store* module provides the functionality for extracting the branches of a given expression for the input program. The *Asymptotics* component of Amber reasons about asymptotic properties of functions and simplifies expressions while preserving their asymptotic behavior. Multiple termination proof rules require the capability of checking whether some property over program variables is eventually invariant. This common requirement is implemented in Amber’s *Invariance* module.

## Evaluation

**Table 1:** 27 programs which are PAST

Program	Amber	Absynth	MGen	LexRSM	KoAT2	ecoimp
2d_bounded_random_walk						
biased_random_walk_const						
biased_random_walk_exp						
biased_random_walk_poly						
binomial_past						
complex_past						
consecutive_bernoulli_trails						
coupon_collector_4						
coupon_collector_5						
dueling_cowboys						
exponential_past_1						
exponential_past_2						
geometric						
geometric_exp						
linear_past_1						
linear_past_2						
nested_loops						
polynomial_past_1						
polynomial_past_2						
sequential_loops						
tortoise_hare_race						
dependent_dist*						
exp_rw_gauss_noise*						
gemoetric_gaussian*						
race_uniform_noise*						
symb_2d_rw*						
uniform_rw_walk*						
Total	23	9	11	12	11	13

**Table 2:** 14 programs which are AST and not necessarily PAST

Program	Amber	LexRSM
fair_in_limit_random_walk		
gambling		
symmetric_2d_random_walk		
symmetric_random_walk_constant_1		
symmetric_random_walk_constant_2		
symmetric_random_walk_exp_1		
symmetric_random_walk_exp_2		
symmetric_random_walk_linear_1		
symmetric_random_walk_linear_2		
symmetric_random_walk_poly_1		
symmetric_random_walk_poly_2		
gaussian_rw_walk*		
laplacian_noise*		
symb_1d_rw*		
Total	12	0

**Table 3:** 9 programs which are not AST

Program	Amber
biased_random_walk_nast_1	
biased_random_walk_nast_2	
biased_random_walk_nast_3	
biased_random_walk_nast_4	
binomial_nast	
polynomial_nast	
binomial_nast_noise*	
symb_nast_1d_rw*	
hypergeo_nast*	
Total	8

**Experimental setup.** Amber and our benchmarks are publicly available at https://github.com/probing-lab/amber. The output of Amber includes the martingale expression and an answer (“Yes”, “No” or “Maybe”) to PAST and AST for the input program. If the answer to (P)AST is definite (“Yes” or “No”), the output additionally contains a witness of the answer. We took all 39 benchmarks from [18] and extended them by 11 new programs to test Amber’s capability to handle symbolic constants and drawing from probability distributions. The 11 new benchmarks are constructed from the 39 original programs, by adding noise drawn from common probability distributions and replacing concrete constants with symbolic ones. As such, we conduct experiments using a total of 50 challenging benchmarks. Further, we compare Amber not only against Absynth and MGen, but also evaluate Amber in comparison to the recent tools LexRSM [7], KoAT2 [16] and ecoimp [17]. Note that MGen can only certify PAST and LexRSM only AST. Moreover, the tools Absynth, KoAT2 and ecoimp mainly aim to find upper bounds on expected costs. Tables 1, 2 and 3 summarize our experimental results, with benchmarks separated into *PAST* (Table 1), *AST* (Table 2), and *not AST* (Table 3). Benchmarks marked with * are part of our 11 new examples. In every table,  () marks a tool (not) being able to certify the respective termination property. Moreover,  symbolizes that a benchmark is out-of-scope for a tool, for instance, due to not supporting some distributions or polynomial arithmetic. All benchmarks have been run on a machine with a 2.6 GHz Intel i7 (Gen 10) processor and 32 GB of RAM and finished within a timeout of 50 s, where most experiments terminated within a few seconds.

**Experimental analysis.** Amber successfully certifies 23 out of the 27 PAST benchmarks (Table 1). Although Absynth, KoAT2 and ecosimp can find expected cost upper bounds for large programs [15–17], they struggle on small programs whose termination is not known a priori. For instance, they struggle when a benchmark probabilistically “chooses” between two polynomials working against each other (one moving the program state away from a termination criterion and one towards it). Our experiments show that Amber handles such cases successfully. MGen supports the continuous uniform distribution and KoAT2 the geometric distribution whose support is infinite. With these two exceptions, Amber is the only tool supporting continuous distributions and distributions with infinite support. To the best of our knowledge, Amber is the first tool certifying PAST supporting both discrete and continuous distributions as well as distributions with finite and infinite support. Amber successfully certifies 12 benchmarks to be AST which are potentially not PAST (Table 2). Whereas the LexRSM tool can certify non-PAST programs to be AST, such programs need to contain subprograms that are PAST [7]. The well-known example of symmetric_1D_random_walk, contained in our benchmarks, does not have a PAST subprogram. Therefore, the LexRSM tool cannot establish AST for it. In contrast, Amber using the *SM-Rule* can handle such programs. To the best of our knowledge, Amber is the first tool capable of certifying non-AST for polynomial probabilistic programs involving drawing from distributions and symbolic constants. Amber is also the first tool automating (non-)AST and (non-)PAST analysis in a unifying manner for such programs.

**Experimental summary.** Tables 1, 2 and 3 demonstrate that (i) Amber outperforms the state-of-the-art in certifying (P)AST, and (ii) amber determines (non-)(P)AST for programs with various distributions and symbolic constants.

## Conclusion

We described Amber, an open-source tool analyzing the termination behavior for polynomial probabilistic programs, in a fully automatic way. Amber computes asymptotic bounding functions and martingale expressions and is the first tool to prove and/or disprove (P)AST in a unifying manner. Amber can analyze continuous, discrete, finitely- and infinitely supported distributions in polynomial probabilistic programs parameterized by symbolic constants. Our experimental comparisons give practical evidence that Amber can (dis)prove (P)AST for a substantially larger class of programs than state-of-the-art tools.

## Data Availability

The tool’s source code, data and benchmarks used in this work are available in Amber’s repository, https://github.com/probing-lab/amber.

## Footnotes

1 https://github.com/lark-parser/lark.

2 https://github.com/diofant/diofant.
